# Next-Generation S3-Level Clinical Practice Guidelines in Periodontology: Methodology, Current Evidence, and Future Directions

**DOI:** 10.3390/dj14010058

**Published:** 2026-01-15

**Authors:** Nada Tawfig Hashim, Ayman Ahmed, Azza A. Abushama, Salma Musa Adam Abduljalil, Bakri Gobara Gismalla, Muhammed Mustahsen Rahman

**Affiliations:** 1 Department of Periodontics, RAK College of Dental Sciences, RAK Medical & Health Sciences University, Ras-AlKhaimah 12973, United Arab Emirates; 2 Department of Oral Rehabilitation, Faculty of Dentistry, University of Khartoum, Khartoum 11115, Sudan; 3 Department of Periodontology and Implantology, Nile University, Khartoum 11115, Sudan; 4 Department of Preventive Dental Sciences (Periodontology), College of Dentistry, Dar Uloom University, Riyadh 13314, Saudi Arabia; a.abdulmahmoud@dau.edu.sa; 5 Department of Periodontology, Faculty of Dentistry, National University, Khartoum 11115, Sudan; salma.m.abduljalil@gmail.com

**Keywords:** periodontitis, S3-level guidelines, clinical practice guideline, evidence-based dentistry, GRADE, endodontics, peri-implantitis, endo-perio lesions, biomarkers, artificial intelligence

## Abstract

**Background**: S3-level clinical practice guidelines represent the highest standard of evidence-based healthcare, integrating systematic reviews, formal evidence grading, and structured expert consensus. In periodontology, current S3-level guidelines provide robust recommendations for the management of stage I–III periodontitis. However, increasing clinical complexity, emerging diagnostic technologies, and the need for patient-centred and implementation-oriented care highlight important gaps that warrant further methodological refinement. **Objective**: This review aims to critically appraise the conceptual foundations, strengths, and limitations of existing S3-level periodontal guidelines and to propose a structured roadmap for the development of next-generation S3 guidance. **Methods**: A narrative and methodological review was conducted focusing on key European S3-level guidelines in periodontology and endodontics, with emphasis on guideline methodology, evidence grading, outcome prioritization, and consensus processes. **Results**: Current S3-level periodontal guidelines demonstrate strong methodological rigor but show limited coverage of stage IV periodontitis, peri-implant diseases, and endo–perio lesions. In addition, emerging domains such as biomarker-based diagnostics, artificial intelligence-assisted decision support, and implementation science are not yet systematically integrated. **Conclusions**: Future S3-level periodontal guidelines should incorporate clinical complexity, patient-reported outcomes, precision diagnostics, digital technologies, and real-world implementation strategies to enhance personalization, transparency, and clinical impact.

## 1. Introduction

Periodontology has undergone a profound transformation over the last two decades, moving from a purely defect- and pocket-based discipline toward a biologically informed, risk-stratified, and patient-centred specialty [1]. The introduction of the 2017 World Workshop classification on periodontitis staging and grading provided a coherent diagnostic scaffold that integrates disease severity, complexity, and risk profile [2]. In parallel, the adoption of S3-level clinical practice guidelines by the European Federation of Periodontology (EFP) for the treatment of stage I–III periodontitis has brought periodontal therapy into alignment with the highest standards of evidence-based medicine [3].

In endodontics, a similar methodological evolution has recently culminated in the publication of the inaugural European Society of Endodontology (ESE) S3-level guideline for the management of pulpal and apical disease [4]. This guideline was built on 14 systematic reviews, a predefined core outcome set, and a structured consensus process, leading to 34 key clinical recommendations framed in transparent, graded language. The endodontic experience demonstrates both the feasibility and the value of fully implementing an S3 approach in a dental specialty. Accordingly, this manuscript is presented as a narrative and methodological review that uses the recently published S3-level endodontic guideline as a methodological comparator to critically appraise existing S3-level periodontal guidance. Rather than duplicating established European Federation of Periodontology (EFP) documents, the review synthesizes their strengths with transferable methodological insights from endodontics, identifies key conceptual and practical gaps, and proposes a rigorous, future-oriented roadmap for the development of next-generation S3-level periodontal clinical practice guidelines.

## 2. Methodological Pillars of S3-Level Clinical Practice Guidelines

### 2.1. The S3-Tier in Guideline Hierarchies

In European medical guideline methodology, S3-level CPGs occupy the highest tier, characterized by [3]:

- A comprehensive set of systematic reviews underpinning every key clinical question.
- Use of structured frameworks such as PICOT (patient/population; intervention; comparator; outcome; time/type).
- Formal use of the Grading of Recommendations Assessment, Development and Evaluation (GRADE) approach to rate the quality of evidence and strength of recommendations.
- A documented, transparent consensus process with clear voting thresholds.
- Explicit declarations of conflict of interest and management of bias.
- The recent ESE S3-level guideline exemplifies this: a steering group, four working groups, 14 systematic reviews, prespecified outcome measures, and a consensus summit that converted evidence profiles into graded recommendations [5].

### 2.2. GRADE and the Evidence-to-Decision Framework

The GRADE methodology provides a structured way to move from evidence to recommendations [5]. It considers not only the internal validity of the studies but also consistency, directness, precision, and publication bias, along with non-evidentiary factors such as patient values, resource use, feasibility, and equity. In the endodontic guideline, this culminated in a clear syntax: “we recommend” (strong), “we suggest” (weak), and “we do not know/may be considered” (open) [4]. The EFP periodontal guideline likewise uses GRADE-based reasoning, but often with a more narrative presentation. A more explicit adoption of the “recommend/suggest/do not know” triad in future periodontal documents would enhance transparency and clinical usability [3].

### 2.3. Core Outcome Sets and Patient-Centred Measures

A striking feature of the ESE guideline is the preliminary development of a core outcome set: tooth survival, resolution of symptoms (pain, swelling), radiographic healing, and oral health-related quality of life were prioritized using a structured, multi-stakeholder Delphi process [4,6].

This is directly relevant to periodontology, where traditional outcomes (probing depth reduction, clinical attachment gain, bleeding on probing) often dominate, while patient-reported outcomes and functional metrics (chewing efficiency, esthetic self-perception, social functioning) are still underrepresented [7].

Future S3-level periodontal guidelines should adopt similarly rigorous processes to rank and standardize both clinical and patient-centred outcomes, including tooth survival, long-term stability, quality of life, and treatment burden.

## 3. Current S3-Level Periodontal Guidelines: Scope and Core Recommendations

The existing S3-level periodontal guidelines for stage I–III periodontitis can be broadly organized into four major domains: diagnosis and classification, risk factor assessment, non-surgical therapy, and surgical and regenerative interventions, all embedded in the framework of lifelong supportive periodontal care [3].

### 3.1. Diagnosis, Staging and Grading

The guideline endorses full-mouth periodontal charting with probing depth, clinical attachment level, bleeding on probing, and radiographic assessment of alveolar bone support. The 2017 classification provides a robust system to stage disease severity (I–IV) and grade progression risk (A–C), incorporating factors such as bone loss–age ratio, smoking, and glycemic control [2]. This structure enhances diagnostic clarity and the ability to stratify patients for tailored therapy [8].

### 3.2. Risk Factor Modification and Behavioural Interventions

A central strength of the current periodontal S3 guidelines is the explicit recognition of modifiable risk factors [3]. Smoking cessation, diabetes control, and personalized oral hygiene instruction form non-negotiable pillars of care. Strong recommendations are given for integrating behavioural and medical counselling into periodontal treatment pathways, in line with the broader concept of periodontitis as a chronic NCD (non-communicable disease) sharing risk factors with cardiovascular disease and diabetes.

### 3.3. Non-Surgical Periodontal Therapy

Non-surgical periodontal therapy (NSPT) is the cornerstone of periodontal care. Recommendations emphasize [3]:

- Thorough supra- and subgingival instrumentation, preferably using a combination of powered and hand instruments.
- Focus on disruption of the subgingival biofilm and calculus removal.
- Reinforcement of patient-performed plaque control.

Adjunctive systemic antibiotics are reserved for specific clinical scenarios with high risk or severe, unresponsive disease. Local antimicrobials and photodynamic therapy receive, at best, weak or conditional recommendations, reflecting evidence that their incremental benefit over well-performed mechanical debridement is modest and context-dependent [9].

### 3.4. Surgical and Regenerative Therapy

For sites with residual deep pockets and/or complex defects following NSPT, the guideline supports flap surgery to gain access for debridement [3]. For deep intrabony defects, regenerative procedures with guided tissue regeneration or enamel matrix derivatives are recommended, supported by a substantial RCT base. Furcation involvement is addressed with resective or regenerative strategies depending on the defect class and tooth strategic value.

### 3.5. Supportive Periodontal Care (SPC)

Supportive periodontal care is correctly highlighted as indispensable for long-term stability. Regular recall intervals, re-evaluation of pocket depths and bleeding indices, reinforcement of oral hygiene, and ongoing risk factor management are central to minimizing recurrence and tooth loss. Evidence clearly demonstrates that the absence of SPC is associated with significantly increased risk of disease progression and tooth loss, even after initially successful therapy [3].

## 4. Lessons from the S3 Endodontic Guideline for Periodontology

The ESE S3-level guideline provides several methodological features that could strengthen future periodontal CPGs [4].

### 4.1. Transparent Strength-of-Recommendation Language

The endodontic guideline repeatedly uses: “we recommend to”, “we suggest to/we suggest not to”, and “we do not know/may be considered”, with explicit notation of evidence quality (high to very low) and rationale [4]. This tight coupling of recommendation strength with evidence certainty improves clinical interpretability and highlights research gaps. A similar discipline in periodontal guideline language would be beneficial; for example, differentiating clearly between strong endorsement of subgingival instrumentation and weak, context-dependent support for adjunctive lasers or probiotics [9,10].

### 4.2. Explicit Acknowledgment of Uncertainty

In several areas—particularly vital pulp therapy choices, regenerative procedures for immature teeth, and endodontic tissue engineering—the ESE guideline openly states “we do not know” and classifies the recommendation as “open”. This honesty about uncertainty is scientifically sound and ethically important, preventing premature overuse of under-evidenced interventions [4]. Periodontology would benefit from similarly explicit statements, particularly around relatively new interventions such as specific host-modulating agents, advanced platelet concentrates, or minimally invasive surgical approaches where long-term comparative evidence is still limited [11].

### 4.3. Balanced View on Adjunctive Technologies

The endodontic guideline critically appraises adjunctive disinfection methods, such as photodynamic therapy, ozone, and lasers, and concludes that they should not be used routinely because comparative outcome evidence does not demonstrate superiority over standard irrigation protocols [4].

Periodontology faces parallel controversies around lasers, photobiomodulation, and various adjunctive technologies. A similarly rigorous, explicitly comparative approach in future periodontal reviews would help distinguish between promising innovations and costly add-ons with marginal benefit [12].

## 5. Critical Appraisal of Current S3 Periodontal Guidance

### 5.1. Strengths

The existing S3 periodontal guideline has several clear strengths [3]:

- It is firmly grounded in systematic review evidence and GRADE reasoning.
- It integrates risk factor modification and behavioral change as central to periodontal care.
- It provides a coherent, step-wise therapeutic pathway from NSPT to surgical and regenerative therapy.
- It highlights the indispensability of maintenance and long-term monitoring.

These features have already improved the consistency and quality of periodontal care across many settings.

### 5.2. Conceptual and Practical Gaps

Despite its strengths, several gaps emerge when the guideline is viewed through a future-focused, S3-level lens:

- Stage IV periodontitis and functional rehabilitation
  Guidance for complex cases involving tooth migration, occlusal dysfunction, and major masticatory impairment is less detailed than for stage I–III disease, despite their high burden and complexity [13,14,15].
- Peri-implant diseases
  Peri-implant mucositis and peri-implantitis now represent a major clinical challenge, often managed by periodontists [16], yet they are not covered in the same integrated S3 framework.
- Endo–perio lesions
  Combined periodontal–endodontic lesions are biologically and clinically intertwined, but guidelines are fragmented between specialties [14]. The new S3-level endodontic work explicitly excludes endo–perio lesions from its remit and calls for dedicated future projects, creating an opportunity for joint guideline development.
- Biomarkers and precision diagnostics
  Despite extensive literature on salivary, serum, and crevicular biomarkers [17,18,19], there is no structured integration of biomarker-based decision making into current recommendations [20,21,22].
- Artificial intelligence and digital workflows
  Radiographic AI, digital periodontal charting, and risk-calculation tools are rapidly emerging, but are not yet reflected in guideline pathways [23,24,25,26].
- Implementation science and health systems
  Real-world adoption, barriers, cost–effectiveness, and health inequalities receive limited explicit attention [27], although they are crucial for translating guideline recommendations into population-level gains.

Taken together, existing S3-level periodontal guidelines represent a major methodological achievement, providing evidence-based and consensus-driven recommendations for the management of periodontitis. Their strengths include rigorous evidence appraisal, structured recommendation grading, and clear therapeutic pathways for stage I–III disease. However, their scope remains limited with respect to advanced disease complexity, peri-implant conditions, interdisciplinary scenarios, emerging diagnostic technologies, and real-world implementation challenges. These limitations form the basis for the proposed next-generation S3-level periodontal guidelines discussed in the following section.

## 6. Toward Next-Generation S3 Periodontal Guidelines: A Proposed Roadmap

**Figure 1:**
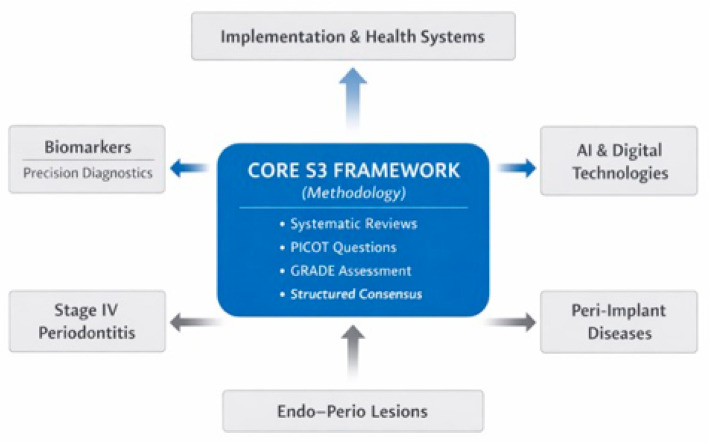
Conceptual structure of a next-generation S3-level clinical practice guideline in periodontology. The figure illustrates the proposed architecture of a next-generation S3-level periodontal guideline. The central core represents established S3 methodology, including systematic reviews, PICOT-based questions, GRADE assessment, and structured consensus. Surrounding this foundation are expanded clinical domains requiring dedicated guidance, including Stage IV periodontitis, peri-implant diseases, and endo–perio lesions. Additional modules incorporate biomarker-driven precision diagnostics, artificial intelligence-assisted decision support, and implementation and health-system integration, highlighting the interrelationships between evidence generation, clinical decision-making, and real-world application. The figure was created by the authors using Microsoft PowerPoint.

To respond to these limitations while preserving the strengths of the current framework, future S3-level periodontal guidelines should expand and deepen their scope in several directions (Figure 1).

Section 6 is structured in two complementary parts. Section 6.1, Section 6.2 and Section 6.3 describe key clinical areas where current S3-level periodontal guidelines remain incomplete and require expansion. Section 6.4, Section 6.5 and Section 6.6 address cross-cutting strategic components that enable next-generation S3 guideline development, including precision diagnostics, digital technologies, and implementation science.

### 6.1. The Need for Dedicated S3-Level Guidance for Stage IV Periodontitis

Stage IV periodontitis presents a clinical picture that goes beyond inflammatory destruction and requires a coordinated, multidisciplinary strategy. These patients often exhibit severe attachment loss, pathologic tooth migration, posterior bite collapse, mobility, and compromised masticatory function [28]. Current periodontal guidelines do not fully capture the clinical decision points relevant to these advanced cases. A future S3-level guideline must therefore translate evidence into practical, scenario-based decision pathways. To support this, Table 1 summarizes the common clinical presentations in Stage IV disease and the corresponding management options that a guideline could standardize.

A future S3-level guideline should not simply state that Stage IV periodontitis requires complex care. Instead, it must clearly define when to preserve teeth, when to realign teeth, when to regenerate, and when to replace teeth. The decision table above reflects what a guideline should provide:

- Clinical scenarios that practitioners commonly face.
- The most evidence-supported primary treatment choice.
- A secondary option when prognosis or systemic factors make the first option unsuitable.
- Transparent criteria that influence decision-making.

This structured, scenario-based approach mirrors the clarity and logical grading system used in the ESE S3 endodontic guideline, which emphasizes transparency, patient-centred reasoning, and explicit acknowledgment of clinical uncertainty. In Stage IV periodontitis, treatment is no longer limited to controlling disease activity. It becomes a process of rebuilding function, stabilizing occlusion, preserving esthetics, and optimizing quality of life.

A dedicated S3-level guideline would therefore offer a unified framework that integrates:

- Periodontal therapy
- Orthodontic correction
- Occlusal rehabilitation
- Regenerative surgery
- Prosthetic reconstruction
- Implant therapy where indicated
- Long-term supportive care

Such a structured guideline would significantly improve consistency of care, reduce treatment variability, and support personalized, evidence-driven decisions in this highly complex patient group.

### 6.2. The Need for an S3-Level Guideline for Peri-Implant Diseases

Peri-implant diseases have become a major global challenge as implant therapy expands. Patients with a history of periodontitis face significantly higher risk of peri-implant mucositis and peri-implantitis, yet current clinical guidance remains heterogeneous, technique-driven, and inconsistently implemented [29]. Unlike periodontitis—where structured, evidence-based pathways exist—peri-implant disease management still varies widely across clinicians and regions [30].

A dedicated S3-level peri-implant guideline would provide standardized, evidence-based recommendations covering diagnosis, risk assessment, nonsurgical and surgical therapy, and structured maintenance. Because peri-implantitis shares immune, microbial, and risk-factor pathways with periodontitis [31], such a guideline must be developed in parallel with periodontal updates to ensure biological and conceptual consistency [3,32]. To illustrate what a future S3 guideline should offer, Table 2 outlines clear clinical scenarios and corresponding decision pathways.

A future S3-level guideline for peri-implant diseases must go beyond procedure-based recommendations and provide structured decision frameworks that clinicians can easily apply. The table above illustrates what such guidance should include:

1. Scenario-specific pathways

Peri-implant mucositis, early peri-implantitis, advanced vertical defects, horizontal defects, esthetic-zone complications, and medically compromised patients each require tailored approaches.

2. Clear rationale for each treatment choice
  Clinicians must understand why a given option is preferred:
  mucositis is reversible, regenerative therapy is ideal for contained vertical defects,
  and resective approaches suit horizontal bone loss.

3. Evidence-based alternatives

When prognosis is limited or when biological or systemic factors restrict treatment, the guideline should clearly state alternative pathways, including conservative strategies or explantation.

4. Decision criteria that mirror the GRADE approach
  The algorithm should reflect transparent reasoning:

- Defect morphology
- Implant surface characteristics
- Keratinized tissue width
- Systemic health
- Esthetic demands
- Patient preference and compliance

These are precisely the types of contextual modifiers that S3-level guidelines are expected to address.

Why This Matters for Future S3 Guidelines

Peri-implant disease is now one of the most rapidly increasing clinical problems in dentistry [32]. Unlike periodontitis—where S3 guidelines already exist—peri-implant disease lacks a unified, international evidence-based standard.A comprehensive S3 guideline would:

- Reduce treatment variability
- Improve long-term implant survival
- Provide clarity on when to preserve versus remove implants
- Integrate new technologies (i.e., air-powder devices, laser-assisted detoxification, probiotics, photodynamic therapy)
- Standardize definitions of success, failure, and recurrence
- Align periodontal and implant-related disease management under a single conceptual model

Such a guideline would fill a major global gap and significantly enhance patient outcomes.

### 6.3. Joint S3-Level Guideline on Endo–Perio Lesions

Endo–perio lesions represent one of the most diagnostically challenging and biologically intertwined conditions in oral medicine [33]. Their complexity lies in the fact that pulpal and periodontal diseases share anatomical pathways (apical foramina, lateral canals, accessory canals, dentinal tubules) and microbial interactions, often leading to overlapping clinical presentations. Despite this, endodontic and periodontal guidelines have traditionally addressed these conditions separately, resulting in inconsistent diagnostic criteria, treatment sequencing, and outcome monitoring [34].

The recent ESE S3-level endodontic guideline has already highlighted the need for dedicated guidance on endo–perio lesions, as these conditions were explicitly excluded from its scope and recognized as requiring separate, interdisciplinary evidence development [3]. A joint EFP–ESE S3-level guideline would harmonize terminologies, unify diagnostic pathways, and standardize treatment strategies across both specialties.

To illustrate how such a guideline could function, Table 3 provides structured, scenario-based decision pathways.

A future joint EFP–ESE S3-level guideline should provide clinicians with a standardized, step-by-step framework for diagnosing and managing endo–perio lesions. The decision table above reflects a structure that mirrors the clarity and logical rigor seen in the ESE S3-level endodontic guideline. Below are key elements such a guideline must include:

1. Standardized diagnostic criteria

- Clear differentiation between:
- Primary endodontic lesions
- Primary periodontal lesions
- True combined lesions
- Root fractures and developmental anomalies

This prevents misdiagnosis and ensures correct treatment sequencing.

2. Clear treatment sequencing

The guideline must explicitly outline which specialty intervenes first based on lesion origin.

The principle remains consistent:

- If the pulp is infected → endodontic treatment first
- If periodontal breakdown precedes pulpal involvement → periodontal therapy first
- If both are present → endodontics first, then periodontal regeneration

This ensures inflammation is controlled internally before addressing external tissue loss.

3. Evidence-based use of imaging

Periapical radiographs remain essential.

CBCT should be used only when diagnosis is uncertain or when differentiating vertical fractures or complex defects.

4. Integrated outcome assessment

Future S3 guidelines should include:

- Tooth survival
- Pocket depth resolution
- Radiographic healing of periapical tissues
- Regeneration of periodontal defects
- Patient-reported outcomes such as comfort, chewing ability, and satisfaction

These combined metrics reflect the dual nature of endo–perio pathology.

5. Interdisciplinary collaboration

True endo–perio lesions require coordinated care between:

- Periodontists
- Endodontists
- Radiologists
- Restorative dentists

An S3-level guideline would create a common language and eliminate the “silo problem” that currently exists between the specialties.

A joint S3-level guideline for endo–perio lesions is not only necessary but long overdue. Such a guideline would unify diagnostic standards, optimize treatment sequencing, leverage appropriate imaging, and combine outcome measures across two biologically connected domains. By doing so, it would elevate the consistency, predictability, and long-term success of care for one of the most misunderstood and mismanaged categories of dental lesions.

While the development of a joint EFP–ESE S3-level guideline on endo–perio lesions would represent a major advance, achieving interdisciplinary consensus is not without challenges. Differences in diagnostic traditions, treatment sequencing preferences, outcome prioritization, and specialty-specific terminology may complicate guideline development. However, both the EFP and ESE have demonstrated the feasibility of overcoming such barriers through structured methodologies, including predefined PICOT questions, independent systematic review teams, transparent GRADE-based evidence-to-decision frameworks, and formal consensus conferences with explicit voting thresholds. The successful development of S3-level guidelines in both periodontology and endodontics provides a robust blueprint for interdisciplinary collaboration. Applying these established consensus models within a joint working group would allow harmonization of perspectives while preserving methodological rigor and clinical relevance.

### 6.4. Incorporation of Biomarkers and Precision Medicine

Although current periodontal S3 guidelines rely primarily on clinical measures such as probing depth, clinical attachment level, and bleeding on probing, advancements in molecular diagnostics now make it possible to evaluate inflammatory activity and tissue breakdown at a biochemical level [3]. Salivary and crevicular biomarkers—including IL-1β, MMPs, RANKL/OPG ratios, and miRNAs—have demonstrated promising diagnostic and prognostic value in predicting disease activity, treatment responsiveness, and risk of recurrence [35]. However, these markers are not yet integrated into clinical guidelines. A future S3-level guideline should therefore define how, when, and why biomarkers should be used to support clinical decisions (Table 4). Doing so would shift periodontal care toward a precision-medicine model in which treatment intensity and recall intervals are individualized rather than uniformly applied.

A future S3-level guideline must move beyond traditional clinical measures and incorporate biological activity as part of periodontal diagnosis and monitoring. Biomarkers allow clinicians to detect inflammation before structural damage becomes clinically evident, to determine which patients are more likely to progress rapidly, and to evaluate whether therapy has effectively suppressed destructive host responses.

1. Diagnostic and prognostic value

Markers such as IL-1β, MMP-8, and MMP-9 reliably indicate active periodontal destruction, while RANKL/OPG ratios better reflect bone metabolism and can predict sites prone to future bone loss [36,37]. Incorporating these into a guideline would provide clinicians with objective signals that complement probing measurements, which are inherently technique-sensitive.

2. Thresholds that change clinical decisions

Future guidelines should specify biologically meaningful thresholds. For example:

- High IL-1β or MMP-8 levels may justify intensified nonsurgical therapy
- Elevated RANKL/OPG ratios may indicate the need for regenerative strategies or closer monitoring.
- Persistent biomarker elevation after therapy may signal residual disease activity requiring re-instrumentation or adjunctive treatment.

3. Biomarker-based recall and maintenance

One of the most valuable applications of biomarkers will be in designing personalized recall intervals:

- Stable biomarker profile → 4–6-month recall
- Elevated biomarkers → 2–3-month recall
- Markedly elevated biomarkers → re-treatment or surgical intervention.

This represents a major shift from current guidelines, which apply recall intervals uniformly.

4. Supporting precision periodontology

The integration of biomarker data will enable clinicians to build patient-specific periodontal profiles that incorporate:

- Biological activity
- Microbial patterns
- Systemic risk factors
- Genetic predisposition
- Treatment responsiveness

Such profiles will allow clinicians to tailor treatment and maintenance schedules in a way that reduces tooth loss, prevents recurrence, and improves quality of life—aligning periodontology with the broader movement toward precision medicine.

Biomarkers offer a scientifically robust and clinically meaningful pathway toward individualized periodontal care [38,39]. A future S3-level guideline should rigorously evaluate their diagnostic thresholds, clarify how biomarker profiles should alter treatment choices, and define how they can inform recall intervals. By embedding molecular diagnostics within a structured S3 framework, periodontology can evolve from a predominantly clinical discipline into one driven equally by objective biological indicators and personalized therapeutic planning.

### 6.5. Integration of AI and Digital Technologies

Artificial intelligence (AI) and digital periodontal technologies are rapidly transforming diagnostic precision, risk assessment, and treatment planning. AI-driven algorithms can quantify bone loss with remarkable reproducibility, detect early pathological changes on radiographs, automate periodontal charting, and generate individualized risk profiles [40]. Yet despite these advances, AI remains absent from current S3-level periodontal guidelines.

A future guideline must therefore provide a structured, evidence-based framework describing when AI should be used, how its outputs should be interpreted, and how clinicians can integrate these tools ethically and safely into practice. To illustrate this, Table 5 outlines potential AI applications and the corresponding clinical decisions a guideline could standardize.

Artificial intelligence has the potential to profoundly reshape periodontal diagnostics and risk assessment, but its integration into clinical guidelines must be cautious, evidence-based, and ethically grounded.

1. AI as a diagnostic adjunct—not a replacement

AI systems can detect early bone loss, subtle changes in periodontal support, or apical pathology with a level of precision that exceeds human reproducibility. However, they cannot interpret symptoms, patient history, or clinical nuance. Future guidelines must therefore emphasize that AI enhances—but does not replace—expert clinical judgment.

2. Defining when AI adds value

An S3-level guideline should specify circumstances where AI improves clinical accuracy:

- When bone loss progression is too subtle for visual detection
- When charting consistency is essential (research, audits, long-term monitoring)
- In complex peri-implant cases where bone morphology is irregular
- When risk stratification requires combining multiple datasets (systemic factors, biomarkers, radiographs)

Such clarity prevents unnecessary reliance on AI while ensuring clinicians benefit from situations where AI demonstrably improves outcomes.

3. Ethical and practical considerations

AI adoption carries important responsibilities:

- Data privacy: Use of patient radiographs and digital records must comply with international data protection standards.
- Transparency: Algorithms should disclose how predictions are made, avoiding “black box” decision-making.
- Bias mitigation: AI models trained on limited populations may incorrectly classify disease in underrepresented groups.
- Clinician oversight: The clinician remains accountable for all diagnostic and therapeutic decisions, regardless of AI input.

Guidelines must explicitly articulate these standards to ensure safe and ethical integration.

4. The role of AI in precision periodontology

When combined with biomarkers, digital charting, and systemic health data, AI becomes a powerful tool for:

- Predicting rapid progressors
- Personalizing recall intervals
- Optimizing treatment sequencing
- Monitoring subtle tissue changes
- Improving long-term maintenance outcomes

This aligns directly with the movement toward precision medicine, in which biologically relevant information and individualized risk profiles guide treatment intensity.

AI and digital technologies offer an unprecedented opportunity to modernize periodontal care. A future S3-level guideline should therefore rigorously evaluate the diagnostic accuracy, clinical usefulness, safety, and ethical implications of AI tools. By defining when and how AI should be used—and by maintaining clinician authority over all final decisions—periodontology can leverage technological innovation without compromising clinical integrity.

### 6.6. Implementation, Equity, and Health-Policy Integration

The scientific strength of S3-level guidelines only translates into improved oral-health outcomes when they are implemented effectively across diverse clinical and socio-economic settings. While current periodontal guidelines focus primarily on diagnosis and treatment pathways, they offer limited direction on *how* recommendations should be integrated into real-world practice—especially in low-resource environments or among vulnerable populations.

A future S3-level periodontal guideline must therefore incorporate principles from implementation science, health equity, and health-policy planning, ensuring that evidence-based care is not only scientifically rigorous but also feasible, accessible, and equitable. This requires structured strategies for clinician education, support for practice change, assessment of economic barriers, and targeted interventions for populations at highest risk of periodontal disease progression.

Concrete implementation strategies are essential for translating S3-level recommendations into daily clinical practice. Clinician education could include modular continuing professional development (CPD) programs aligned with major guideline domains (e.g., diagnosis and staging, non-surgical therapy, regenerative and surgical decision-making), interactive case-based workshops, and digital learning platforms incorporating self-assessment modules and audit tools. In addition, chairside decision-support systems—such as digital periodontal charting software linked to guideline-based algorithms, risk calculators integrating clinical parameters, biomarkers, and systemic risk factors, and simplified treatment flowcharts embedded within electronic dental records—could support real-time, evidence-informed clinical decision-making during patient care.

Pilot implementation of these strategies in academic centers and large clinical networks would allow assessment of feasibility, clinician acceptance, and impact on treatment consistency and patient outcomes, thereby informing iterative refinement and wider dissemination of future S3-level periodontal guidelines.

To support this, Table 6 outlines actionable domains for implementation within a future S3 framework.

Implementation science provides the operational bridge between guideline recommendations and actual clinical practice. For periodontal care—where prevention, behaviour change, and long-term maintenance are critical—implementation becomes just as important as the clinical evidence itself [39].

1. Embedding guideline recommendations in daily practice

Educational initiatives, decision-support tools, and standardized charting systems can help clinicians consistently apply guideline recommendations. Without these supports, even the strongest evidence may fail to reach patients.

2. Evaluating outcomes through audit and feedback

Future S3 guidelines should specify recommended audit cycles, key performance indicators (e.g., BOP rates, maintenance adherence, tooth retention), and mechanisms for feedback. This encourages accountability, practice improvement, and alignment with international standards.

3. Enhancing accessibility in low-resource environments

Periodontal disease disproportionately affects populations in low- and middle-income countries [41], where periodontal care is often unaffordable or unavailable. A next-generation S3 guideline should:

- Highlight cost-effective therapeutic strategies
- Provide simplified non-surgical treatment protocols
- Address challenges such as limited instrumentation, lack of imaging technologies, or reduced specialist access

This ensures that guidelines do not unintentionally widen global disparities in periodontal health.

4. Addressing vulnerable populations and health inequalities

Groups facing socioeconomic hardship, chronic medical conditions, or limited health literacy carry a higher periodontal burden yet receive less preventive care. A future S3 guideline must therefore recommend:

- Adjusted recall intervals
- Simplified oral hygiene instructions
- Integrated care with medical providers (e.g., diabetes clinics)
- Community-level preventive programs

This aligns periodontal care with WHO principles on equity and universal health coverage [42].

5. Integrating periodontal care into health policy and NCD frameworks

Periodontitis shares risk factors with major non-communicable diseases—diabetes, cardiovascular disease, obesity, and smoking [43,44,45]. A next-generation S3 guideline should encourage policymakers to:

- Embed periodontal screening within NCD programs
- Include periodontal treatment in insurance coverage
- Support national prevention and public-awareness strategies

This elevates periodontology from a specialty-level focus to a core component of public oral-health policy.

Future S3-level periodontal guidelines must extend beyond evidence synthesis and treatment recommendations to address the realities of delivering care across diverse populations and health systems. By incorporating implementation strategies, equity considerations, and health-policy integration, next-generation guidelines can ensure that scientifically proven interventions translate into real-world improvements in periodontal health. This multidimensional approach—linking evidence, practice, and public health—is essential for maximizing the global impact of periodontology in the era of chronic disease management and precision medicine.

## 7. Conclusions

Next-generation S3-level periodontal guidelines must evolve beyond the classical clinical pathway of diagnosis, non-surgical therapy, surgery, and maintenance. As this review illustrates, periodontology now stands at a critical point where scientific progress, digital transformation, and global health realities demand a more comprehensive, interdisciplinary, and equity-focused framework.

While current S3 guidelines offer strong, evidence-based recommendations for the management of stage I–III periodontitis, significant clinical gaps remain. Stage IV periodontitis requires explicit, scenario-driven decision pathways that integrate periodontal therapy with orthodontic correction, occlusal rehabilitation, regenerative approaches, and prosthetic reconstruction. Similarly, the global rise of peri-implant diseases highlights the urgency for an S3-level implant-specific guideline to standardize diagnosis, decontamination strategies, regenerative indications, and surgical decision-making. The management of endo–perio lesions—long divided between specialties—would benefit greatly from a joint EFP–ESE S3 guideline that harmonizes terminology, diagnostic criteria, sequencing of care, and shared outcome measures.

Equally important is the integration of emerging scientific and technological advances. Biomarker-based diagnostics offer a pathway toward precision periodontology by enabling individualized risk profiling, biologically informed treatment decisions, and personalized maintenance intervals. Artificial intelligence, digital charting, and radiographic interpretation tools have the potential to enhance diagnostic accuracy and risk prediction—provided that their clinical value, limitations, and ethical considerations are rigorously evaluated within an S3 framework.

Finally, even the most methodologically robust guideline will remain incomplete without clear strategies for implementation, equity, and health-system integration. Future S3 guidelines should embed tools for clinician education, audit and feedback, cost-effective care models, and tailored approaches for vulnerable and medically complex populations. By aligning periodontal care with broader non-communicable disease frameworks and public health policy, the guidelines can ensure that advanced scientific knowledge translates into real-world improvements in oral health outcomes.

The next generation of S3-level periodontal guidelines should be more than a technical update—they should represent a transformative blueprint for modern periodontal care. By integrating interdisciplinary decision pathways, biomarker science, digital innovation, and public-health principles, periodontology can move decisively toward a model of care that is predictive, personalized, equitable, and globally applicable. This multidimensional evolution is essential for meeting the needs of contemporary clinical practice and for improving periodontal health at both individual and population levels.

## Figures and Tables

**Table 1:** Clear Decision Pathways for Common Stage IV Periodontitis Scenarios.

Clinical Scenario	Primary Treatment Approach	Why This Approach?	Alternative Option	Key Factors Influencing Decision
1. Pathologic tooth migration (spacing, flaring, extrusion)	Control inflammation → orthodontic realignment → definitive restorative work	Tooth alignment restores function and esthetics; orthodontics only stable after inflammation control	Extraction + implant where prognosis is hopeless	Bone support, patient esthetic needs, systemic health, compliance
2. Loss of posterior support and bite collapse	Stabilize remaining teeth → re-establish posterior support (implants or fixed prostheses)	Posterior stability protects anterior teeth and restores mastication	Removable prosthesis if implants are contraindicated	Bone volume, patient systemic status, affordability
3. Grade III mobility in strategic teeth	Splinting + occlusal adjustment + periodontal surgery	Stabilization reduces traumatic forces and allows healing	Extraction if mobility persists despite therapy	Crown–root ratio, mobility pattern, occlusal forces
4. Deep intrabony defects in esthetic zone	Regenerative periodontal surgery (GTR/EMD)	Regeneration preserves esthetics and tooth structure	Extraction + implant if defect morphology is unfavorable	Defect walls, esthetic risk, patient preference
5. Masticatory dysfunction (chewing difficulty, TMJ strain)	Occlusal rehabilitation → orthodontics if required → definitive prosthodontics	Restores comfort, function, and TMJ balance	Shortened dental arch for medically complex patients	Age, TMJ condition, prosthetic space
6. Severe disease in medically compromised patients	Conservative therapy + risk control + simple restorative solutions	These patients heal more slowly; aim for stability not complexity	Extraction + conservative prosthesis	Diabetes control, immune status, medications
7. Hopeless teeth with adequate bone for implants	Planned extractions → staged implant therapy → full-arch reconstruction	Predictable functional and esthetic outcomes	Periodontal prosthesis when implants are not feasible	Implant risk profile, bone anatomy, expectations

**Table 2:** Decision Pathway Matrix for Future S3-Level Guidelines on Peri-Implant Diseases.

Clinical Scenario	Primary Management Approach	Why This Approach?	Alternative Option	Key Factors Influencing Decision
1. Peri-implant mucositis (no bone loss)	Mechanical debridement + biofilm control + patient hygiene reinforcement	Early mucosal inflammation is fully reversible	Adjunctive antiseptics if bleeding persists	Patient hygiene, smoking, prosthetic design
2. Early peri-implantitis (shallow bone loss, moderate bleeding/pus)	Nonsurgical decontamination + targeted antimicrobial strategies	In early disease, detoxification can arrest progression	Minimally invasive surgical access if pockets persist	Defect morphology, implant surface roughness
3. Moderate to advanced peri-implantitis with vertical bone defects	Regenerative peri-implant surgery (bone graft + membrane)	Regeneration restores lost bone and reduces pocket depth	Combined regenerative + resective surgery	Defect configuration (3-wall vs. 1-wall), implant position
4. Horizontal bone loss or non-contained defects	Resective surgery + implantoplasty + pocket reduction	Resective therapy better suits non-regenerative defects	Explantation if significant mobility or hopeless anatomy	Width of keratinized mucosa, prosthetic demands
5. Peri-implantitis in esthetic zone	Regenerative approach with soft-tissue augmentation	Regeneration and soft-tissue grafting preserve esthetics	Explantation + delayed implant if severe	Smile line, tissue biotype, patient esthetic expectations
6. Peri-implantitis in medically compromised patients (e.g., uncontrolled diabetes)	Conservative debridement + risk-factor stabilization	Reduces microbial load while avoiding surgical trauma	Removal of implant in uncontrolled high-risk cases	Healing capacity, HbA1c, medications
7. Severely advanced peri-implantitis with implant mobility	Explantation → site decontamination → staged re-implantation or alternative prosthesis	Mobility indicates implant failure; removal prevents further destruction	Fixed or removable prosthesis without implant replacement	Bone availability, systemic factors, patient preference

**Table 3:** Clear Decision Pathways for Endo–Perio Lesions in a Future Joint S3-Level Guideline.

Clinical Scenario	Primary Diagnosis	Recommended Treatment Sequence	Why This Sequence?	Key Diagnostic Tools
1. Primary endodontic lesion with secondary periodontal involvement (deep isolated pocket, sinus tract tracing to apex)	Pulpal origin	Endodontic treatment first → reassess periodontal healing	Treating the source (infected pulp) often leads to resolution of the periodontal component	Pulp vitality tests, probing pattern, sinus tract tracing, periapical X-ray
2. Primary periodontal lesion with secondary endodontic involvement (generalized bone loss, periodontal recession, late pulpal necrosis)	Periodontal origin	Periodontal therapy first → endodontic treatment only if pulp becomes necrotic	Periodontal inflammation is the initial driver; pulpal involvement is secondary and often occurs late	Full-mouth periodontal charting, radiographs showing generalized bone loss
3. True combined lesion (concurrent pulpal necrosis and advanced localized bone loss reaching apex)	Dual origin	Endodontic treatment first, followed by periodontal regenerative or resective therapy	Eliminating intraradicular infection improves prognosis of regenerative or periodontal surgery	CBCT if needed, periapical radiographs, vitality testing, defect morphology evaluation
4. Vertical root fracture mimicking endo–perio lesion	Structural defect	Extraction (most cases) or root resection (if strategic root remains)	Fracture provides non-healing pathway; periodontal and endodontic therapy alone cannot resolve	CBCT, transillumination, probing pattern (narrow deep pocket)
5. Endo–perio lesion in esthetic zone	Depends on origin	Endodontic treatment first, then minimally invasive periodontal regeneration	Protects esthetics by maintaining root structure and supporting soft tissue healing	CBCT for labial plate integrity, periapical imaging
6. Endo–perio lesion in medically compromised patients	Mixed or unclear	Conservative endodontic therapy + simplified periodontal therapy	Reduced surgical healing capacity necessitates minimally invasive approach	Vitality, probing, medical risk assessment

**Table 4:** How Biomarkers Could Be Incorporated into a Future S3-Level Periodontal Guideline.

Clinical Need	Relevant Biomarkers	Interpretation & Thresholds	Clinical Decision Triggered	Rationale
1. Early detection of active disease	IL-1β, MMP-8, MMP-9	Elevated levels indicate ongoing connective-tissue breakdown	Initiate or intensify non-surgical therapy	Identifies inflammation before deep pockets form
2. Assessing risk of rapid progression	RANKL/OPG ratio, IL-6, TNF-α	High RANKL/OPG ratio suggests active bone resorption	Shorter recall intervals (1–3 months)	Predicts sites likely to lose attachment
3. Monitoring response to therapy	MMP-8, salivary IL-1β	Declining levels = positive response; persistently high = incomplete resolution	Re-evaluate instrumentation, consider adjunctive therapy	Guides need for additional intervention
4. Determining recall frequency	Composite biomarker panel	Stable or low levels = maintenance every 4–6 months; high levels = 2–3 month intervals	Personalized supportive periodontal care	Moves away from a “one-size-fits-all” recall model
5. Identifying relapse or early recurrence	miRNA panels (e.g., miR-146a, miR-155)	Upregulation suggests reactivation of inflammation	Early re-entry for professional cleaning	Detects recurrence before clinical deterioration
6. Estimating prognosis for questionable teeth	RANKL/OPG + MMP-8 combo	Persistently elevated biomarkers despite therapy	Consider regenerative surgery or extraction	Adds biological justification to prognostic decisions

**Table 5:** Potential Roles of AI in a Future S3-Level Periodontal Guideline.

AI Application	What AI Provides	When It Adds Value	Clinical Decision Supported	Key Considerations
1. AI-based radiographic bone loss analysis	Automated and reproducible measurement of bone levels on periapical or panoramic radiographs	When clinician estimates vary; when monitoring subtle changes over time	Diagnose early bone loss; identify progression sites; tailor therapy	Image quality, calibration, clinician verification
2. Automated digital periodontal charting	Objective probing depth, recession, and BOP mapping via optical or sensor-based systems	For patients requiring frequent monitoring or large datasets	Support diagnosis, track healing, and standardize maintenance timelines	Calibration with manual probing; cost–benefit analysis
3. AI-based risk prediction models	Individualized risk scores combining clinical data, radiographs, biomarkers, and systemic factors	When stratifying patients into low, moderate, or high progression risk	Adjust recall intervals; decide between conservative vs. intensive therapy	Transparency of algorithm; bias control; patient-specific variables
4. AI-supported diagnosis of peri-implant diseases	Automated detection of peri-implant bone loss and mucosal inflammation	In early stages where visual detection is difficult	Early intervention for mucositis or implant decontamination	Implant design variability; surface reflectivity
5. AI-enhanced decision-support systems	Treatment suggestions based on aggregated evidence and patient profile	When clinicians face complex Stage III/IV cases	Guide sequencing of therapy (NSPT, surgery, regeneration)	Clinician oversight essential; avoid over-reliance
6. AI-assisted CBCT interpretation	Detection of bone defects, root fractures, and anatomical variations	When conventional imaging is inconclusive	Plan regenerative surgery, manage endo–perio lesions	Radiation dose, over-detection risks

**Table 6:** Implementation and Equity Priorities for a Future S3-Level Periodontal Guideline.

Domain	Implementation Need	Practical Applications	How This Improves Equity and Outcomes
1. Clinical implementation strategies	Consistent adoption of guideline-based care	Training programs, chairside decision-support tools, standardized periodontal charting templates	Reduces variability in care; supports clinicians in all settings
2. Audit and feedback systems	Monitoring adherence and patient outcomes	Regular audit cycles, digital dashboards, feedback to clinicians	Identifies gaps, improves quality, supports continuous improvement
3. Access and affordability	Care must be feasible in low- and middle-income (LMIC) contexts	Prioritizing cost-effective therapies, simplified maintenance models, subsidies or community programs	Ensures evidence-based care is not limited to high-income populations
4. Infrastructure and resource constraints	Many clinics lack advanced equipment	Scalable recommendations, low-tech alternatives for diagnostics, flexible treatment pathways	Allows guideline uptake regardless of technology level
5. Vulnerable and high-risk populations	Patients with NCDs, disabilities, low socioeconomic status, or limited access	Tailored recall intervals, simplified self-care protocols, integration with medical services	Addresses disparities in disease burden and outcomes
6. Health-policy integration	Long-term sustainability of periodontal care	National guidelines, insurance coverage policies, integration with NCD prevention frameworks	Embeds periodontal care in broader public health systems
7. Patient empowerment	Informed, engaged patients have better outcomes	Culturally adapted educational materials, multilingual resources, tele-periodontology	Improves self-care, adherence, and early disease detection

## Data Availability

The original contributions presented in this study are included in the article. Further inquiries can be directed to the corresponding author.
